# Supplementary material for: Rapid Antibacterial Activity Assessment of Chimeric Lysins
Source: Int J Mol Sci. 2024 Feb 19;25(4):2430. doi: 10.3390/ijms25042430 (PMC10888538; doi:10.3390/ijms25042430)
Supplement: Supplementary file 1 [file ijms-25-02430-s001.zip › ijms-2862092-supplementary.pdf]

International Journal of
Molecular Sciences

MDPI

Supplementary Information

Rapid Antibacterial Activity Assessment of Chimeric Lysins

Jin-Mi Park [1,2,3], Jun-Hyun Kim [1,2,3], Gun Kim [2,4], Hun-Ju Sim [2,4], Sun-Min Ahn [1,], Kang-Seuk Choi [2,5,*] and Hyuk-Joon Kwon [1,2,3,*]

1 Laboratory of Poultry Medicine, College of Veterinary Medicine, Seoul National University, Seoul 08826, Republic of Korea; niji17@snu.ac.kr (J.-M.P.); loupgarouhs@snu.ac.kr (J.-H.K.); vicky.ahn@snu.ac.kr (S.-M.A.)
2 College of Veterinary Medicine and BK21 for Veterinary Science, Seoul National University, Seoul 08826, Republic of Korea; smilesssss@snu.ac.kr (G.K.); gjswn0302@snu.ac.kr (H.-J.S.)
3 GeNiner Inc., Seoul 08826, Republic of Korea
4 Laboratory of Pharmacology, Research Institute for Veterinary Science, College of Veterinary Medicine, Seoul National University, Seoul 08826, Republic of Korea
5 Laboratory of Avian Diseases, College of Veterinary Medicine, Seoul National University, Seoul 08826, Republic of Korea
* Correspondence: kchoi0608@snu.ac.kr (K.-S.C.); kwonhj01@snu.ac.kr (H.-J.K.); Tel.: +82-2-880-1266 (K.-S.C. & H.-J.K.)

Table S1. Information of SH3 domains used in this study.

Abbreviated name	Sequence identity to K_{SH3} (%)	Genbank Protein Full name	Genbank accession No.	Amino acid Region	Amino acid Length
L25(K_{SH3})	100	LysK [*Staphylococcus phage* P4W]	AFN38929	409-495	87
$ClyC_{SH3}$	94.2	Lysin [*Staphylococcus phage* PALS_1]	QDJ97474.1	123-209	87
vB_{SH3}	93.1	N-acetylmuramoyl-L-alanine amidase [*Staphylococcus phage* vB_Sau_Clo6]	ARM69344.1	409-495	87
$tras14_{SH3}$	40.2	endolysin [*Staphylococcus phage* trsa14]	AWH13066.1	395-481	87
$ClyO_{SH3}$	11.4	PlySs2 family phage lysin [*Streptococcus suis*]	WP_024417665.1	162-245	84
Lsp_{SH3}	9.1	Lysostaphin [*Staphylococcus simulans*]	WP_013012297.1	369-452	84
$2638A_{SH3}$	6.8	endolysin [*Staphylococcus phage* 2638A]	YP_239818.1	401-486	86
2958_{SH3}	4.5	endolysin [*Staphylococcus phage* phi2958PVL]	YP_002268027.1	405-484	80

Table S2. Information of lysostaphin and chimeric lysins used in this study.

Protein name	Genbank accession No.	Amino acid Region	Amino acid Length	Reference
Lysostaphin	WP_013012297.1	207-452	246	[34]
Lsp		207-368	162	
ClyC	AGO49867.1	1-171	171	[24]
	InsertVD	172-173	2	
	QDJ97474.1	102-209	108	
ClyO	WP_258391128.1	1-157	157	[33]
	InsertG	158	1	
	WP_024417665.1 (R195C)	146-245	100	

Table S3. Analyses of genetic traits related to expression levels of chimeric lysins.

Chimeric lysin	RI	Frequency of amino acid (%)		Translation stalling codon pair[c]	Free energy[d] (kcal/mol)
		Low expression-related amino acid[a]/codon[b]	high expression-related amino acid		
ALS2-dA-L21	**1.00**	52.3 (11/21)/ 14.2 (3/21)	52.3% (11/21)	PP, PG	-14.4/ -213.2
ALS2-dA-L24	**0.35**	50.0 (12/24)/12.5 (3/24)	50.0% (12/24)	PP, PG	-16.3/-213.6
ALS2-dA-L25	**0.95**	52.0 (13/25)/16.0 (4/25)	52.0% (13/25)	PP, PG	-16.5/-219.2
ALS2-dA-L26	**0.93**	50.0 (13/26)/15.3 (4/26)	50.0% (13/26)	PP, PG	-15.1/-214.8
ALS2-dA-L27	**0.52**	48.1 (13/27)/14.8 (4/27)	48.1% (13/27)	PP, PG	-15.2/-213.9
ALS2-dA-L28	**0.76**	46.4 (13/28)/14.2 (4/28)	46.4% (13/28)	PP, PG	-16.3/-216.0
ALS2-dA-L31	**0.46**	45.1 (14/31)/12.9 (4/31)	45.1% (14/31)	PP, PG	-23.4/-222.7
ALS2-dA-L34	**0.23**	47.0 (16/34)/11.7 (4/34)	47.0% (16/34)	PP, PG	-25.6/-221.7
ALS2-dA-L35	**0.57**	48.5 (17/35)/14.2 (5/35)	48.5% (17/35)	PP, PG	-28.5/-227.0
ALS2-dA-L38	**0.33**	44.7 (17/38)/13.1 (5/38)	44.7% (17/38)	PP, PG	-29.6/-227.8
ALS2-dA-L25	0.32	54.0 (47/87) 19.5 (17/87)	11.5% (10/87)	PP, PG	-85.7/-219.2
ALS2-dA-ClyC$_{SH3}$	0.40	54.0 (47/87) 16.0 (14/87)	11.5% (10/87)	-	-66.0/-203.4
ALS2-dA-vB$_{SH3}$	0.36	55.1 (48/87) 18.3 (16/87)	11.5% (10/87)	-	-64.3/-197.8
ALS2-dA-tras14$_{SH3}$	0.57	48.2 (42/87) 16.0 (14/87)	18.4% (16/87)	-	-76.9/-210.1
ALS2-dA-ClyO$_{SH3}$	0.64	44.0 (37/84) 8.3 (7/84)	15.4% (13/84)	-	-81.7/-208.3
ALS2-dA-Lsp$_{SH3}$	0.91	46.4 (39/84) 15.4 (13/84)	19.0% (16/84)	-	-59.6/-187.5
ALS2-dA-2638A$_{SH3}$	1.00	40.6 (35/86) 9.3 (8/86)	30.2% (26/86)	-	-54.4/-187.3
ALS2-dA-2958$_{SH3}$	0.09	50.0 (40/80) 12.5 (10/80)	10.0% (8/80)	-	-56.1/-184.9
ALS2-dA-L25	0.29	50.6% (115/227)/16.7% (38/227)	13.2% (30/227)	PP, PG SH3	-1.3/-219.2
Lysostaphin	1.00	46.9% (116/247)/15.4% (38/247)	19.0% (47/247)	-	-4.6/-214.4
Lsp-ClyC$_{SH3}$	0.10	49.6% (124/250)/15.6% (39/250)	16.4% (41/250)	-	-4.6/-224.8
ClyC	0.17	47.6% (134/281)/11.7% (33/281)	19.9% (56/281)	-	-3.6/-241.8
ClyO	2.08	46.9% (121/258)/13.2% (34/258)	15.9% (41/258)	-	-10.0/-245.5

[a] The frequency of L, S, R, A, P, G, C, W, and I in linker, SH3 domain, insert gene and initial~16codon.
[b] The frequency of AGG (R), CGU (R), CGA (R), AGU (S), GGU (G), AGA (R), UGU (C), CGG (R), CCC (P), AUA (I) in linker, SH3 domain and complete coding region.
[c] Ribosome stalling codon pairs: RR (CGG-AGG, AGG-CGG, CGA-CGG, CGG-AGG), PP (CCA-CCC, CCG-CCA, CCT-CCG, CCG-CCG, CCT-CCT, CCA-CCG), KR (AAG-AGG), LR (CTG-AGG), PG (CCG-GGT, CCG-GGG), VG (GTG-GGA), and QD (CAG-GAT).
[d] Predicted free energy of mRNA secondary structure of chimeric lysin by RNA fold. Partial regions: linker, SH3 domain, and first 16-dodons.

Table S4. PCR primers used for amplification of 10 linker candidates

Primer name	Sequence (5' -3')	Usage
P T7-F	TCGAAATTAATACGACTCACTATAG	IVTC/TL amplification for all
T term-R	GAGGGGATCCAGATCTTGGGAT	
P_gCHAP-L-F	CTTTAAGAAGGAGATATACATATGG-CATCATCTTTTAATCACC	Linker & SH3 domain SOE-PCR for all
P_gCHAP-L-R	GTGATTAAAA-GATGATGCCATATGTATATCTCCTTCTTAAAG	
KSH3_T-L-F	GTTGCATGGGGTGTTTTCAAAAAGGGTCATCATCAC-CATCAC	Linker & SOE-PCR for all
KSH3_T-L-R	GTGATGGTGATGATGACCCTTTTT-GAAAACACCCCATGCAAC	
gCHAP_L21-L-F	GCTATGCATTCATCCATTCTGCATCTACTCCGGCAAC	SOE-PCR for linker21
gCHAP_L21-L-R	GTTGCCGGAGTAGATGCAGAATGGATGAATGCATAGC	
gCHAP_L24-L-F	GCTATGCATTCATCCATAAAACTTCTTCTGCATCTAC	SOE-PCR for linker24
gCHAP_L24-L-R	GTAGATGCAGAAGAAGTTTTATGGATGAATGCATAGC	
gCHAP_L25-L-F	GCTATGCATTCATCCATGGTAAAACTTCTTCTGCATC	SOE-PCR for linker25
gCHAP_L25-L-R	GATGCAGAAGAAGTTTTACCATGGATGAATGCATAGC	
gCHAP_L26-L-F	GCTATGCATTCATCCATGACGGTAAAACTTCTTCTGC	SOE-PCR for linker26
gCHAP_L26-L-R	GCAGAAGAAGTTTTACCGTCATGGATGAATGCATAGC	
gCHAP_L27-L-F	GCTATGCATTCATCCATAAAGACGGTAAAACTTCTTC	SOE-PCR for linker27
gCHAP_L27-L-R	GAAGAAGTTTTACCGTCTTTATGGATGAATGCATAGC	
gCHAP_L28-L-F	GCTATGCATTCATCCATGTTAAAGACGGTAAAACTTC	SOE-PCR for linker28
gCHAP_L28-L-R	GAAGTTTTACCGTCTTTAACATGGATGAATGCATAGC	
gCHAP_L31-L-F	GCTATGCATTCATCCATTCTACTGTCGTTAAAGAC	SOE-PCR for linker31
gCHAP_L31-L-R	GTCTTTAACGACAGTAGAATGGATGAATGCATAGC	
gCHAP_L34-L-F	GCTATGCATTCATCCATACTTCTTCTTCTACTGTCG	SOE-PCR for linker34
gCHAP_L34-L-R	CGACAGTAGAAGAAGAAGTATGGATGAATGCATAGC	
gCHAP_L35-L-F	GCTATGCATTCATCCATGGTACTTCTTCTTCTACTG	SOE-PCR for linker35
gCHAP_L35-L-R	CAGTAGAAGAAGAAGTACCATGGATGAATGCATAGC	
gCHAP_L38-L-F	GCTATGCATTCATCCATATGGACAAGGGTACTTCTTC	SOE-PCR for linker38
gCHAP_L38-L-R	GAAGAAGTACCCTTGTCCATATGGATGAATGCATAGC	

*Underlined letters indicate the sequence of gCHAP fragments.

Table S5. PCR primers used for amplification 8 SH3 domain linker

Primer name	Sequence (5' -3')	Usage
ALS2-dA-ClyC$_{SH3}$-L-F	GAAAAACCAGTACGGTACTTGGTACAAACCGGAATCG	SOE-PCR for ALS2-dA-ClyC$_{SH3}$
ALS2-dA-ClyC$_{SH3}$-L-R	CGATTCCGGTTTGTACCAAGTACCGTACTGGTTTTTC	
ALS2-dA-vB$_{SH3}$-L-F	GAAAAACCAGTACGGTACTTGGTACAAACCGGAAAACG	SOE-PCR for ALS2-dA-vB$_{SH3}$
ALS2-dA-vB$_{SH3}$-L-R	CGTTTTCCGGTTTGTACCAAGTACCGTACTGGTTTTTC	
ALS2-dA-tras14$_{SH3}$-L-F	GAAAAACCAGTACGGTACTTACTACATGGAAGAAAGC	SOE-PCR for ALS2-dA-tras14$_{SH3}$
ALS2-dA-tras14$_{SH3}$-L-R	GCTTTCTTCCATGTAGTAAGTACCGTACTGGTTTTTC	
ALS2-dA-ClyO$_{SH3}$-L-F	GAAAAACCAGTACGGTACTCGTTCCTATCGCGAGAC	SOE-PCR for ALS2-dA-ClyO$_{SH3}$
ALS2-dA-ClyO$_{SH3}$-L-R	GTCTCGCGATAGGAACGAGTACCGTACTGGTTTTTC	
ALS2-dA-Lsp$_{SH3}$-L-F	GAAAAACCAGTACGGTACTCTGTATAAATCTGAATCTG	SOE-PCR for ALS2-dA-Lsp$_{SH3}$
ALS2-dA-Lsp$_{SH3}$-L-R	CAGATTCAGATTTATACAGAGTACCGTACTGGTTTTTC	
ALS2-dA-2638A$_{SH3}$-L-F	GAAAAACCAGTACGGTACTTGGTATAAAGCTGAACATG	SOE-PCR for ALS2-dA-2638A$_{SH3}$
ALS2-dA-2638A$_{SH3}$-L-R	CATGTTCAGCTTTATACCAAGTACCGTACTGGTTTTTC	
ALS2-dA-2958$_{SH3}$-L-F	GAAAAACCAGTACGGTACTGGAAATTATACCGTCGC	SOE-PCR for ALS2-dA-2958$_{SH3}$
ALS2-dA-2958$_{SH3}$-L-R	GCGACGGTATAATTTCCAGTACCGTACTGGTTTTTC	

*Underlined letters indicate the sequence of gCHAP fragments.

Table S6. PCR primers used for amplification of various chimeric lysins

Primer name	Sequence (5' -3')	Usage
P_Lsp-L-F	CTTTAAGAAGGAGATATACATatgGCGGCAACGCATGAAC	SOE-PCR for Lysostaphin
P_Lsp-L-R	GTTCATGCGTTGCCGCcatATGTATATCTCCTTCTTAAAG	
Lsp_T-L-F	CTGTGGGGTACTATCAAAAAGGGTCATCATCACCATC	SOE-PCR for Lysostaphin & Lsp_ClyC$_{SH3}$
Lsp_T-L-R	GATGGTGATGATGACCCTTTTTGATAGTACCCCACAG	
P_ClyC-L-F	CTTTAAGAAGGAGATATACATATGCAAGCAAAACTAAC	SOE-PCR for ClyC
P_ClyC-L-R	GTTAGTTTTGCTTGCATATGTATATCTCCTTCTTAAAG	
ClyC_T-L-F	CCTGGGGAACATTCAAGAAGGGTCATCATCACCATC	
ClyC_T-L-R	GATGGTGATGATGACCCTTCTTGAATGTTCCCCAGG	
P_ClyO-L-F	CTTTAAGAAGGAGATATACATATGGCACTGCCTAAAACGG	SOE-PCR for ClyO
P_ClyO-L-R	CCGTTTTAGGCAGTGCCATATGTATATCTCCTTCTTAAAG	
ClyO_T-L-F	CTTGGGGTACATTTAAAAAGGGTCATCATCACCATC	
ClyO_T-L-R	GATGGTGATGATGACCCTTTTTAAATGTACCCCAAG	
Lsp_ClyC-L-F	GAAAACTAACAAATATGGTACTTGGTACAAACCGGAATCG	SOE-PCR for Lsp_ClyC$_{SH3}$
Lsp_ClyC-L-R	CGATTCCGGTTTGTACCAAGTACCATATTTGTTAGTTTTC	

*small case letters indicate nucleotides added artificially.

Table S7. PCR primers used for amplification of pET101/D/TOPO vector

Primer name	Sequence (5' -3')	Usage
gCHAP_CACC-F	caccATGGCATCATCTTTTAATC	ALS2-dA-L25 amplification
K$_{SH3}$-R	TTTGAAAACACCCCATGCAAC	ALS2-dA-L25 amplification
ClyC$_{SH3}$-R	CTTGAATGTTCCCCAGGCAAC	ALS2-dA-ClyC$_{SH3}$ amplification

*small case letters indicate nucleotides added artificially.

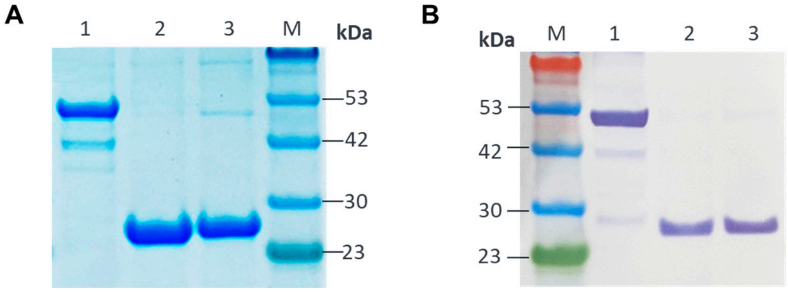

Figure S1. SDS-PAGE and Western blotting of *E. coli* expressed chimeric lysins. **(A)** SDS-PAGE analysis of purified protein using 4-12% polyacrylamide gel. Lane M: protein molecular weight marker; Lane 1: purified protein of ALS2; Lane 2: purified protein of ALS2-dA-L25; Lane 3: ALS2-dA-ClyC$_{SH3}$. For SDS-PAGE analysis, 5 μL of each purified protein, (B) Western blot analysis of expressed protein by six histidine antibody. The molecular weight of the protein inserted into the pET/D/101 vector is approximately 52.4kDa and 28.31kDa. Lane M: protein molecular weight marker (PM2700 ExcelBand™ 3-color Broad Range Protein Marker); Lane 1: Protein expressed ALS2; Lane 2: Protein expressed ALS2-dA-L25; Lane 3: Protein expressed ALS2-dA-ClyC$_{SH3}$. For western blot analysis, 20 μL of each induced protein.

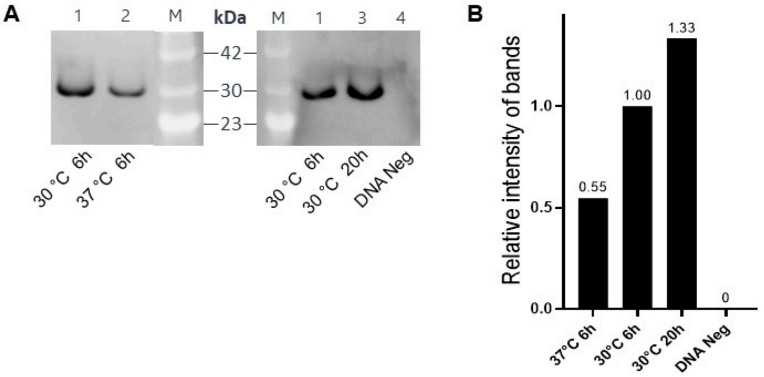

Figure S2. Comparison of expression levels of Lsp-ClyC$_{SH3}$ in different temperature and incubation times. **(A)** Western blotting of Lsp-Cly$_{SH3}$ using anti-histidine antibody and ECL. Lane M: protein molecular weight marker; Lane 1: Expressed at 30°C for 6h; Lane 2: Expressed at 37°C for 6h; Lane 3: Expressed at 30°C for 20h; Lane 4: DNA-negative sample. **(B)** The relative intensity of the Lsp-ClyC$_{SH3}$ protein bands. The ratio of protein expression compared to Lsp-ClyC$_{SH3}$ expressed at 30°C for 6h was measured using Image J 1.53
